# Frequency of lower extremity artery disease in type 2 diabetic patients using pulse oximetry and the ankle-brachial index

**DOI:** 10.7150/ijms.58907

**Published:** 2021-05-27

**Authors:** Mosquera-Fernández Abián, Balboa-Barreiro Vanesa, Bellido-Guerrero Diego, González-Sagrado Manuel, Vale-Carrodeguas Maria, Veiga-Seijo Raquel, González-Martín Cristina

**Affiliations:** 1 Department of Health Sciences. Faculty of Nursing and Podiatry. University of A Coruña (UDC) Ferrol Campus, 15471, Ferrol, Spain.; 2 Rheumatology and Public Health Research Group, Nursing Research and Health Care, Biomedical Research Institute of A Coruña (INIBIC), Complejo Hospitalario Universitario de A Coruña (CHUAC), SERGAS, University of Coruña (UDC), As Xubias 84, 15006 A Coruña, Spain.; 3 Coordinator of the Endocrinology and Nutrition Unit Ferrol Naval Hospital (Ferrol Health Area). Department of Health Sciences. University of A Coruña (UDC). Ferrol Campus, 15471, Ferrol, Spain.; 4 Research Support Unit. Rio Hortega University Hospital. Dulzaina 2. 47012, Valladolid, Spain.; 5 Nurse at the Ferrol University Hospital Complex (Ferrol Health Area). Avda. de la Residencia s/n. 15405 Ferrol, Spain.

**Keywords:** diabetes, pulse oximetry, lower extremity artery disease

## Abstract

**Objectives:** To determine the of undiagnosed lower extremity artery disease using the pulse oximetry in a type 2 diabetic population sample.

**Methods:** Observational, cross-sectional, descriptive study that included 594 type 2 diabetic patients, with no previous history of lower extremity artery disease. Medical history, physical examination, determination of the ankle-brachial index (portable Doppler) and measurement of oxygen saturation in upper and lower extremities (pulse oximeter) were performed.

**Results:** Frequency of lower extremity artery disease determined by ankle-brachial index was 18.4%. No significant correlations were detected between oxygen saturation and the ankle-brachial index except for the relationship between ankle-brachial index vs. oxygen saturation at 30 cm lower limb elevation vs. the supine position at no elevation (0 cm) in subjects under the age of 40. Pulse oximetry showed little diagnostic value in the screening of lower extremity artery disease. A relationship between lower extremity artery disease and age has been found. Its diagnosis was associated with a lower body mass index and lower systolic blood pressure in the lower extremities and higher in the upper extremities.

**Conclusions:** We conclude that pulse oximetry is not useful in the screening for asymptomatic lower extremity artery disease in type 2 diabetics.

## Introduction

Excess glucose in the blood causes dysfunction and failure of various organs, but particularly blood vessels and nerves. Its evolution is usually asymptomatic (it is estimated that between 30% and 50% of patients are not diagnosed), which means that at the time of diagnosis it is already possible to verify a certain degree of vascular deterioration. Currently, diabetes mellitus (DM) is diagnosed according to the plasma glucose value (fasting or 2 hours after an oral glucose overload) or according to the glycated hemoglobin value (≥6.5%) 1.

Recently, a prevalence of diagnosed DM in the United States of 8.2% has been estimated, calculating that it could reach 10.5% (more than 34 million people) if undiagnosed cases are added to those diagnosed 2. Globally, DM continues to increase throughout the world unevenly depending on the country and affected population. In fact, it is expected that by 2040 there will be 642 million people with DM around the world, further increasing the associated health expenditure, which was already estimated at 673 billion dollars in 2015 2-3.

On the other hand, PAD is the leading cause of morbidity and mortality in diabetics. It is a progressive, systemic disease that causes arterial stenosis mainly in the lower limb. It is a good marker of systemic atherosclerosis and an important predictor of cardiovascular morbidity and mortality. Although the etiology of atherosclerosis is multifactorial, DM constitutes one of its main risk factors 4. Its prevalence increases significantly with age, in such a way that from the 5.6% detected in people between 38 and 59 years of age it goes to 15.9-29% between 60 and 69 years 5-7. Currently, it affects more than 200 million people around the world, being one of the most prevalent pathologies and with the highest morbidity and mortality in the world that causes approximately 37.3 billion dollars related to cardiovascular disease and DM 8-10. Epidemiological studies such as REACH-E, FRENA and AIRVAG analyze the impact of PAD in different vascular territories and affirm that the distribution of coronary heart disease is broader than cerebrovascular disease and that those with PAD in addition to having poorer control they tend to present new ischemic episodes in another vascular territory 11-13. In the AIRVAG study, for example, 21% of the individuals with PAD also had asymptomatic vascular involvement in another vascular territory, and in the VITAMIN study, the prevalence of PAD was 21.3% in non-diabetics 14.

Both known and unknown PAD are more frequent in diabetics than in the general population. In this sense, published data differ markedly and can even vary among diabetics followed in primary and specialized care. As an example, in primary care centers of Catalonia the prevalence of unknown PAD reported according to the Ankle-Brachial Index (ABI) was of 21.5% among type 2 diabetics with a mean age of 65.3 years.

However, in consultations of Endocrinology throughout Spain among diabetics (type 1 and type 2) with an average age of 59 years, the prevalence of PAD was 37.3% 15-16. Among diabetics aged 50-85 years seen in internal medicine consultations (MERITO study), the prevalence of unknown PAD was 26.2% and in diabetics older than 70 years followed in specialized care (internal medicine, geriatrics and endocrinology) the prevalence of PAD was 60.6% (in 41.2% of asymptomatic cases), which raised up to 71% based on ABI 17-18.

ABI is a bloodless, simple, cheap and reproducible test with high specificity to identify an obstruction in the arteries of the lower limb, making the diagnosis of lower extremity artery disease (LEAD) possible. It is routinely used in disease screening, allowing the identification of asymptomatic patients in whom early detection would make it possible to initiate adequate treatment, control of cardiovascular risk factors and even modify risk stratification. The American Diabetes Association (ADA) recommends its use in the initial screening for PAD among those who present signs or symptoms and asymptomatic cases with clinical suspicion 19-20. However, ABI has sometimes shown a low sensitivity, it has not been able to detect asymptomatic patients and falsely high values have been obtained in the presence of arterial calcification or stenosis of the subclavian artery 21-24. For this reason, and because ABI measurement requires prior training and is time-consuming is often not performed during routine daily practice.

The definitive method or “gold standard” for diagnosing PAD is contrast angiography because of its ability to provide detailed information about arterial anatomy. However, contrast angiography is invasive and carries some risk.

On the other hand, although ABI is not the “gold standard” its diagnosis, it is routinely used in daily practice as it is a cheap, simple and painless method.

An ABI> 0.90 has a sensitivity of 75% and a specificity of 86% for the diagnosis of LEAD 25. Its sensitivity is lower in diabetic patients or patients with terminal chronic kidney disease (CKD) due to calcification of the middle layer 26. Patients with borderline ABI (0.90-1.00) have to undergo additional diagnostic tests.

Hence, there is a need for non-invasive LEAD diagnostic techniques, easy to perform, cost-effective and able to detect asymptomatic LEAD. In this sense, pulse oximetry has been proposed as a screening tool for LEAD with contradictory results. It has been suggested that it could have greater sensitivity and as much accuracy as ABI 27-29, but on the other hand previous research did not find significant differences in pulse oximetry based on the presence of carotid plaques depending on pathological ABI values or carotid intima-media thickness 29.

Therefore, the main goal of this study is to determine the frequency of undiagnosed LEAD using the pulse oximetry in a type 2 diabetic population sample. The secondary outcome of the study is to:

- Application of pulse oximetry in LEAD screening;
- Detect LEAD using ABI in the sample studied;
- Study the relationship between LEAD and different epidemiological-clinical variables;
- Determine the correlation between ABI and oxygen saturation (SaO_2_).

## Experimental Section

A cross-sectional observational study was carried out in the outpatient clinics of the Endocrinology and Nutrition Service of the Hospital Naval of Ferrol (Ferrol Health Area) from May 2016 to June 2018.The inclusion criteria were type 2 diabetic patients with no previous diagnosis of LEAD who attended these consultations during the study period and who signed the informed consent. The exclusion criteria were Raynaud disease, vasculitis and claudication symptoms. The sample size was 594 patients, which allowed for calculation of the prevalence and 95% confidence interval (CI) of LEAD with a precision of 3% for a significance level of 0.05. The patients who participated in the study were included consecutively. Furthermore, all participants received nutritional counseling. For this purpose, there was a nurse in charge who did this counseling.

### Measurements

The participants were recruited by the Endocrinology and Nutrition specialists. Both, the information gathering and the examinations were carried out in the Service's own external consultations. Sociodemographic variables (age, sex), anthropometric variables (body mass index (BMI), waist circumference, HbA1c**,** dyslipemia, LDL-cholesterol, total cholesterol**,** arterial hypertension, smoking habits, symptoms of neuropathy through the diapason and clinical variables systolic blood pressure (BP) in the lower right and lower left extremity in the supine position. Systolic and diastolic BP were collected from all participants recruited in a sitting and supine position as well as the presence of peripheral pulses). All patients had ABI measured using a bidirectional portable Doppler (Mini Doppler, model ES-100X® Hadeco Inc. Kawasaki, Japan) at 8 MHz. With the patient in the supine position, the systolic BP of both arms was determined using an appropriate pressure cuff that was placed above the antecubital fossa. Systolic BP was measured at the level of the pedial dorsal and posterior tibial artery. ABI was calculated for each lower limb.

ABI values lower than 0.90 30 were considered pathological and diagnostic of LEAD. In addition, two measurements of SaO_2_ were made with a digital pulse oximeter (Onyx® 9500, Nonin Medical Inc. Plymouth, MN, USA), one at the great toe of each foot in the supine position (horizontal) and another measurement with the limb elevated 30 cm. SaO_2_ was also recorded at both index fingers (upper limb). Regarding the results of pulse oximetry, the SaO_2_ difference in the great toes compared to index fingers (toe to finger TTF) and the difference in SaO_2_ of the great toes with the lower limb elevated 30 cm (T30) compared to the supine position at no elevation (T0) were measured.

Thus, according to previous research 28-29 it was considered pathological:

- A decrease in SaO_2_ greater than 2% at the great toes measured in the supine position (no elevation) compared to the highest SaO_2_ at both index fingers.
- A decrease in SaO_2_ greater than 2% at the great toes with the lower limb elevated 30 cm (T30) compared to the measurement at the great toe of the same foot in the supine position at no elevation (T0).

The usefulness of each of these measurements for LEAD screening was evaluated compared to ABI.

A calibrated thermohygrometer was used in the room where the measurements were taken. The parameters considered as normal were: humidity of 10-80% and temperature of 20-26 ºC. Between each participant, these parameters were checked to be the same in all the exploration.

The statistical analysis was performed with the SPSS version 24.0 program. For the study of the diagnostic tests, the EPIDAT version 3.1 program for epidemiological analysis of tabulated data was used. A descriptive analysis of all the parameters analyzed was carried out. The quantitative variables were described as mean ± standard deviation (SD) and their normality established with the Kolmogorov-Smirnov test. The qualitative variables were described by absolute frequencies and percentages. The prevalence of LEAD was determined according to the ABI together with its 95% CI. Differences between patients with or without LEAD were analyzed. The comparison of means was carried out by means of the Student's t test or the Mann-Whitney test, as appropriate. The association of qualitative variables was estimated with the Chi-square statistic. Using the data obtained in the lower limbs, the correlation of TTF and T30-T0 with ABI was determined using Pearson's linear correlation coefficient or Spearman's Rho. The concordance of both indices with the ABI for the diagnosis of LEAD was also determined using the Kappa index. The area under the ROC curve (AUC), and the sensitivity, specificity, predictive values, and likelihood ratios were calculated, along with their 95% CI.

### Ethical-legal aspects

At all times, the ethical standards for the ethical evaluation of epidemiological studies and biomedical research in human subjects were followed. On the other hand, the information regarding the identification of the participants was considered confidential for all purposes so that their identity could not be revealed or disclosed in accordance with the provisions of the Organic Law on Protection of Personal Data. Furthermore, the participants were informed of the purpose of the study and their informed consent was obtained respecting their right to privacy ABI and digital pulse oximetry are both non-invasive harmless screening procedures tests.

The study was approved by the Institutional Ethics Committee (CE 07/2016). All participants signed informed consents.

Privacy of patients as well as data confidentiality were protected at all times.

## Results

A total of 594 patients (1188 lower limbs) were included. The general characteristics of the sample according to sex are shown in Table 1. Mean age was 62.9 ± 11.0 years. 266 women (44.8%) and 328 men (55.2%) were studied. The BMI presented a mean of 30.7 ± 6.3 kg/m^2^, with a 49.7% and a 34.1% classified as obese and overweight respectively. 36.4% of the sample smoked and 18.2% were ex-smokers. The mean ABI was 1.07 ± 0.25 in the right lower limb and 1.04 ± 0.24 in the left lower limb, with the prevalence of LEAD (ABI <0.90) of 18.4% (95% CI: 15.1% -21.5%). 8.4% of the sample showed LEAD in both lower limbs. Regarding to the smoking habits, 36.4% smoked, 45.4% didn't smoke and 18.2% ex-smoked.

Systolic BP in the upper limbs, of both (seated and supine position) were significantly higher in the group diagnosed with LEAD, while the BP measured in the lower limbs was significantly lower. There were no differences in brachial diastolic BP. Although there were not significant differences, peripheral pulses were present slightly more often in subjects without diagnosis of LEAD (Table 2). Regarding the maximum value of SaO_2_ at both index fingers and SaO_2_ at both great toes in the supine position and at 30 cm elevation, not statistically significant differences were detected between patients with or without diagnosis of LEAD according to ABI (Table 2).

To assess the correlation of the measurements made with the pulse oximeter and the ABI in the diagnosis of LEAD (TTF and T30-T0). The results obtained for each lower limb were evaluated, comparing them with the ABI results obtained for the same lower limb. ABI was pathological in 159 lower limbs (13.6%). A decrease of more than 2% was observed in the SaO_2_ at the great toe compared to the SaO_2_ at the index finger in 113 (9.8%) lower limbs, and a decrease of more than 2% in the SaO_2_ at the great toe at 30 cm elevation compared to the supine position in 98 (9.1%) lower limbs. Combining these last two criteria, a pathological result was obtained by pulse oximetry in 207 (17.9%) lower limbs. Table 3 shows the concordance, correlation, and validity indices of pulse oximetry with respect to ABI. The TTF and T30-T0 values did not show correlation with the ABI, with zero agreement. Both parameters showed high values of specificity and negative predictive value, but low values of sensitivity and positive predictive value.

The area under the curve (AUC) was 0.525 (95% CI = 0.476-0.573) for TTF and 0.504 (95% CI = 0.454-0.553) for T30-T0. These values were similar regardless of the age and gender of the patients (Table 4).

## Discussion

In the present study, we have found data on mean age and BMI very similar to those obtained by Pellitero et al. (62 ± 7 years and 31.5 kg/m^2^, respectively) 29. The mean BMI of the sample corresponds to type I obesity, being higher in women, as is also reported by the majority of studies carried out among Spanish adults according to the Spanish Society for the Study of Obesity (SEEDO) 31-32. In this sense, the ERICE study describes a higher prevalence of obesity in women, with a difference compared to men that is accentuated after the age of 50 33.

Regarding BP, it has been reported that arterial hypertension is a powerful risk factor for vascular complications such as ischemic heart disease or cerebrovascular disease 34. In Spain, it has been estimated a 33% prevalence of hypertension among the general adult population 35.

In the studied sample, mean values of systolic and diastolic BP are respectively within the range of hypertension according to the classification of the European Societies of Hypertension and Cardiology 36. In the present study, both systolic BP and diastolic BP were higher in men and significantly higher in older patients, which is consistent with the ERICE study on the main cardiovascular risk factors in our country, which indicates that the prevalence of hypertension is considerably higher in men at younger ages (20-44 years) and somewhat higher in women from 65 years of age. In both cases, the prevalence of hypertension increases progressively with age 33.

Regarding the diagnosis of LEAD, it was established using the ABI with a 0.90 cut-off point, since it has shown a high sensitivity and specificity to detect LEAD 8. To this regard, the frequency of LEAD found is lower than the published by other authors 37-38, which could be partially explained by the fact that the studied sample had no diagnosis of cardiovascular disease.

Related to pulse oximetry, it is normally used for medical conditions requiring constant monitoring of blood gases in areas such as intensive care units, emergency medicine and anesthesia. However, in previous studies it has been proposed as an indirect indicator of tissue perfusion used in the treatment of ischemic lesions in lower limbs 39 and as an alternative to ABI in the screening of LEAD 27-28 with contradictory results. In our country, other authors have analyzed its usefulness in the screening of carotid atherosclerosis in type 2 diabetics, comparing this test with carotid ultrasound and ABI 29. In this study, no differences were observed in pulse oximetry based on the presence or absence of carotid plaques evaluated by ultrasound, thus lacking usefulness in the screening of carotid atherosclerosis. More recently Ena et al. found that pulse oximetry in diabetic patients did not have enough sensitivity to detect LEAD 40. Nevertheless, studies outside our country have observed a greater sensitivity of pulse oximetry compared to ABI in type 2 diabetics aiming that the combination of both tests (ABI and pulse oximetry) could increase the sensitivity obtained 41. Similarly, it has been suggested that in patients with symptomatic LEAD pulse oximetry could have as much precision as ABI 42.

Regarding the concordance between pulse oximetry and ABI, we have not detected significant correlations between SaO_2_ values and ABI except in the ABI vs. T30-T0 in patients under 40 years of age in whom a low positive correlation was observed (Spearman's Rho = 0.343; p = 0.02), in agreement with other authors 29, 43. Regarding the differences analyzed, the mean value of SaO_2_ obtained in the supine position (lower limb at no elevation) was 96.4 ± 2.4%, while with the lower limb at 30 cm elevation it was 95.9 ± 2.5%. The mean of the TTF was 0.19 ± 2.3% and -0.45 ± 1.8% for the T30-T0. As in the study by Pellitero et al.^29^ in our case we have also not observed statistically significant differences for the differences considered (TTF and T30-T0) or significant correlations of the SaO_2_ values with the ABI.

### Limitations

One of the main limitations of ABI in diabetic patients is the frequent presence of arterial calcifications in the lower limbs, obtaining abnormally high values, but, on the other hand, diabetic subjects can obtain a great benefit from its measurement. Regarding pulse oximetry, some situations or external factors can cause uncertain results or incorrect readings to be obtained, such as interference with other electrical devices, strong ambient light (fluorescent), a decrease in body temperature, hypotension or vasoconstriction, and presence of dyshemoglobinemia 44.

### Selection biases

Selection biases may arise from the inclusion and exclusion criteria determined for the execution of the study. In this case, no data was collected from participants who refused to participate, this percentage being less than 5%, with which we consider that the representativeness of the sample has not been affected.

### Information biases

Information biases that arise from how the data were obtained can occur.

Variability produced by the type of procedure or test used to perform the exams.

These biases were minimized, to the extent possible, through the establishment of validated questionnaires, calibrated instruments, experienced personnel, and repeated measurements.

### Confusion biases

Due to the absence of variables in the data collection that should have been taken into account for the realization of this study and that are not included due to ignorance of them.

To minimize this bias, a multivariate logistic regression analysis will be performed.

## Conclusions

Pulse oximetry showed little association with ABI in the diagnostic of LEAD. A relationship between LEAD and age has been found. Its diagnosis was associated with a lower body mass index and lower systolic blood pressure in the lower extremities and higher in the upper extremities. We conclude that pulse oximetry is not useful in screening for asymptomatic LEAD in type 2 diabetics.

## Author Contributions

Data collection: A. M.-F.; conception and design of the study: A. M.-F.; D. B.-G.; M G.-S.; M. V-C; anlysis of data V.B.-B.; the writing of the article or the critical review of a substantial part of its content A. M.-F.; D. B.-G.; M G.-S.; V. B.-B.; M.V.-C.; C.G.-M.

## Figures and Tables

**Table 1:** General characteristics of the sample

	Total (n=594)	Men (n=328)	Women (n=266)	p
	Mean ±SD	Mean ±SD	Mean ±SD	
Age (years)	62.9 ± 11.0	62.1 ± 10.5	64 ± 11.6	**0.037**
Body Mass Index (Kg/m^2^)	30.7 ± 6.3	29.8 ± 5.5	31.7 ± 7.1	**<0.001**
Waist circumference (cm)	101.2 ± 14.7	102.1 ± 13.6	100.1 ± 15.9	0.108
HbA1c	8.2 ± 1.7	8.2 ± 1.6	8.2 ± 1.8	0.441
LDL-cholesterol	98.2±35.0	98.7±33.3	99.8±37.4	0.554
Total cholesterol	174.6 ±42.1	171.5 ±42.0	179.1 ±42.2	0.164
**Diapason**				
Left	4.5±2.6	4.2±2.6	4.9±2.5	0.000
Right	4.5±2.6	4.2±2.7	4.9±2.4	0.001
**Systolic BP in LL (mmHg)**				
Right	157.3 ± 39.7	159.6 ± 42	154.6 ± 36.6	0.128
Left	153.1 ± 38.0	155.7 ± 42.1	149.9 ± 32.1	0.061
Systolic brachial BP (seated) (mmHg)	146.1 ± 20.8	145.4 ± 19.9	146.9 ± 22	0.398
Diastolic brachial BP (seated) (mmHg)	79.3 ± 10.2	78.8 ± 10.1	79.8 ± 10.3	0.270
Systolic brachial BP (supine) (mmHg)	148.4 ± 21.3	148.4 ± 21.3	148.2 ± 21.4	0.902
SaO_2_ index finger (%)	96.2 ± 1.9	96.1 ± 1.9	96.4 ± 2.1	0.121
**SaO_2_ index finger (supine) (%)**				
Right	96.2 ± 2.8	96.3 ± 2.7	96.2 ± 3	0.816
Left	96.3 ± 2.7	96.1 ± 2.9	96.5 ± 2.4	0.121
**SaO_2_ LL 30 cm elevation (%)**				
Right	95.5 ± 3.9	95.6 ± 3.2	95.3 ± 4.6	0.397
Left	95.6 ± 3.6	95.5 ± 3.7	95.7 ± 3.3	0.469
**Ankle-Brachial Index**				
Right	1.07 ± 0.25	1.08 ± 0.27	1.05 ± 0.22	0.133
Left	1.04 ± 0.24	1.05 ± 0.27	1.02 ± 0.19	0.126
	**n (%)**	**n (%)**	**n (%)**	**p**
Hypertension	159 (61.4)	92 (60.1)	67 (63.2)	0.617
Dyslipemia	155 (60.1)	89 (58.2)	66 (62.9)	0.450

LL: Lower limbs; BP: blood pressure; SaO_2_: Oxygen saturation. The maximum value was taken in the variables: Systolic brachial BP (seated and supine), Diastolic brachial BP (seated) and SaO_2_ index finger.

**Table 2:** Clinical variables according to the diagnosis of lower extremity artery disease

	LEAD No (n=485)	LEAD Yes (n=109)	p
	Mean ± SD	Mean ± SD	
Age (years)	62.6 ± 10.9	63.5 ± 11.2	0.180
Body Mass Index (kg/m^2^)	31 ± 6.3	30.3 ± 6.4	0.100
Waist circumference (cm)	101.5 ± 14.5	100.7 ± 15.1	0.427
**Systolic BP in LL (mmHg)**			
Right	169.3 ± 39.7	139.3± 32.4	**<0.001**
Left	166.5 ± 38.5	133.2± 27.0	**<0.001**
**Seated brachial BP (mmHg)**			
Systolic	143.5 ± 20.1	149.9± 21.4	**<0.001**
Dyastolic	79.1 ± 9.6	79.6 ± 11.0	0.538
**Supine systolic brachial BP (mmHg)**			
Maximum	144.6 ± 20.3	154.0 ± 21.6	**<0.001**
**Pedial pulse (n (%))**			
Right	320 (89.6%)	220 (92.8%)	0.185
Left	323 (90.7%)	221 (93.2%)	0.275
**Posterior tibial pulse (n (%))**			
Right	305 (85.9%)	215 (90.7%)	0.080
Left	306 (85.7%)	215 (90.7%)	0.069
**SaO_2_ in UL (%)**			
Maximum	96.2 ± 2	96.3 ± 2.1	0.755
**SaO_2_ in LL supine (%)**			
Right	96.3 ± 2.6	96.2 ± 3.0	0.783
Left	96.3 ± 2.7	96.3 ± 2.7	0.781
**SaO_2_ at 30 cm LL elevation (%)**			
Right	95.5 ± 4.1	95.5 ± 3.5	0.841
Left	95.6 ± 3.3	95.5 ± 3.9	0.870
**LDL-cholesterol**	98.2 ± 32.1	98.3 ± 41.6	0.354
**Total cholesterol**	173.5 ± 40.8	177.2 ± 45.5	0.991
**HbA1c**	8.2±1.7	8.3±1.8	0.938
**Hypertension**	117 (64.3)	42 (54.5)	0.141
**Dyslipidemia**	109 (59.9)	46 (60.5)	0.924

LL: lower limb; UL: upper limb; BP: blood pressure; SaO_2_: oxygen saturation; LEAD: lower extremity artery disease; NS: not significant; ABI: Ankle-Brachial Index.

**Table 3:** Concordance, correlation and validity of indices derived from pulse oximetry with ankle-brachial index for the diagnosis of lower extremity artery disease

MEASUREMENT	TTF		T30-T0	
	r	p	r	p
Correlation with ABI	-0.006	NS	0.013	NS
	AUC	95% IC	AUC	95% IC
AUC (95% CI)	0.525	0.476-0.573	0.504	0.454-0.553
	Kappa	95% CI	Kappa	95% CI
Kappa Index	0.033	-0.030-0.095	-0.021	-0.076-0.033
	**% ( 95% CI)**		**% (95% CI)**	
Sensitivity (%)	22.7 (17.6-28.7)		34.9 (28.81-41.67)	
Specificity (%)	84.1 (79.8-87.7)		69.2 (63.8-74.1)	
VPP (%)	49.1 (39.4-58.8)		43.5 (36.2-51.1)	
PPV (%)	61.8 (57.3-66.2)		61.0 (55.8-65.9)	
NPV (%)	1.4 (1.0-2.0)		1.1 (0.9-1.5)	
LHR+ (%)	0.9 (0.8-1.0)		0.9 (0.83-1.06)	
LHR- (%)	22.7 (17.6-28.7)		34.9 (28.81-41.67)	
PPV (%)	87.2 (85.1- 89.3)		87.1 (84.9-89.2)	
NPV (%)	1.3 (0.8-2.1)		0.8 (0.4-1.5)	
LHR+ (%)	0.97 (0.91-1.03)		1.02 (0.97-1.07)	
LHR- (%)	0.03 (-0.03-0.08)		-0.02 (-0.07-0.03)	

AUC: Area under the curve; ABI: Ankle-brachial index; TTF: Toe to finger oxygen saturation difference; T30-T0: Oxygen saturation difference of great toe at 30 cm elevation vs. supine position. NS: not significant. LHH +: Likelihood Ratio +; LHR-: Likelihood Ratio; PPV: positive predictive value; NPV: negative predictive value; CI: confidence interval.

**Table 4:** ROC curve and AUC of the indices derived from pulse oximetry to predict lower extremity artery disease related to the ankle-brachial index. Analysis according to sex and age

ROC CURVE	TTF		T30-T0	
	AUC	95% CI	AUC	95% CI
Global	0.525	0.476-0.573	0.504	0.454-0.553
**Age**				
≤ 65 years	0.569	0.503 - 0.635	0.508	0.439 - 0.577
> 65 years	0.485	0.413 - 0.557	0.520	0.447 - 0.593
**Sex**				
Men	0.554	0.488 - 0.621	0.493	0.425 - 0.562
Women	0.493	0.422 - 0.564	0.516	0.444 - 0.589

TTF: Great toe to index finger oxygen saturation difference; T30-T0: Oxygen saturation difference of great toe at 30 cm elevation vs. supine position; AUC: Area under the curve; CI: confidence interval.
